# Speaking up as an extension of socio-cultural dynamics in hospital settings: a study of staff experiences of speaking up across seven hospitals

**DOI:** 10.1108/JHOM-04-2022-0129

**Published:** 2022-11-15

**Authors:** Antoinette Pavithra, Russell Mannion, Neroli Sunderland, Johanna Westbrook

**Affiliations:** Centre for Health Systems and Safety Research , Australian Institute of Health Innovation , Sydney, Australia; Australian Institute of Health Innovation , Sydney, Australia; Health Services Management Centre , University of Birmingham , Birmingham, UK

**Keywords:** Speaking up, Unprofessional behaviour, Culture change intervention, Organisational change, Hospital employees, Bourdieu

## Abstract

**Purpose:**

The study aimed to understand the significance of how employee personhood and the act of speaking up is shaped by factors such as employees' professional status, length of employment within their hospital sites, age, gender and their ongoing exposure to unprofessional behaviours.

**Design/methodology/approach:**

Responses to a survey by 4,851 staff across seven sites within a hospital network in Australia were analysed to interrogate whether speaking up by hospital employees is influenced by employees' symbolic capital and situated subjecthood (SS). The authors utilised a Bourdieusian lens to interrogate the relationship between the symbolic capital afforded to employees as a function of their professional, personal and psycho-social resources and their self-reported capacity to speak up.

**Findings:**

The findings indicate that employee speaking up behaviours appear to be influenced profoundly by whether they feel empowered or disempowered by ongoing and pre-existing personal and interpersonal factors such as their functional roles, work-based peer and supervisory support and ongoing exposure to discriminatory behaviours.

**Originality/value:**

The findings from this interdisciplinary study provide empirical insights around why culture change interventions within healthcare organisations may be successful in certain contexts for certain staff groups and fail within others.

## Introduction

Healthcare organisational culture is defined as the shared assumptions, values, thinking and beliefs that influence norms and impact individual and group behaviours, practices and dynamics ( Mannion and Davies, 2018 ; Manley and Jackson, 2019 ). Within the literature that examines healthcare organisational culture, concepts such as “safety culture” and “safety climate” are used interchangeably with “culture” to refer to the elements of organisational culture that comprise or enhance (1) the safety and quality of care provided to patients, (2) an atmosphere of mutual trust and psychological safety for staff and (3) the absence of retribution, retaliation, blame, punishment ( Halligan, 2011 ; Pfaff *et al.* , 2009 ).

Culture change interventions have in the recent past gained popularity across healthcare organisations, in response to the prevalence of normalised unprofessional behaviours in healthcare work ( May *et al.* , 2018 ; Jones *et al.* , 2021 ). Unprofessional behaviour is characterised as wrongdoing which spans instances of individual, group and systemic misconduct involving illegal, illegitimate, or immoral behaviours and dynamics within organisations ( Skivenes and Trygstad, 2014 ; Pavithra, 2021 ). Within healthcare organisations, unprofessional behaviours which range from interprofessional incivility to professional misconduct have been found to impact patient safety, staff wellbeing and organisational outcomes ( Douglas *et al.* , 2021 ; Westbrook *et al.* , 2020 ). Within this context, culture change interventions in healthcare aim to modify the normalisation or acceptance of unprofessional behaviours and encourage positive behaviours within an organisation to improve patient care quality and safety and improve psychological safety for staff ( Blenkinsopp *et al.* , 2019 ; Churruca *et al.* , 2022 ; Stacy *et al.* , 2022 ).

A key lever used by culture change interventions involves enhancing employee voice behaviours to promote psychological safety within organisations ( Kakkar and Tangirala, 2018 ; Novak, 2019 ). These interventions include speaking up training and improving graded assertive communication skills to build collective awareness of what behaviours and events may require employee speaking and to also enable employees with the non-technical skills required to disrupt unprofessional behaviours when they occur ( Wong and Ginsburg, 2017 ; McKenzie *et al.* , 2019 ). While constructs invoked by the term “speaking up” vary across multiple bodies of scholarship, there is an inextricable link between existing definitions of speaking up in relation to patient advocacy, individual employee agency and voice and open cultures that encourage error reporting and whistleblowing in healthcare ( Fagan *et al.* , 2016 ). When negative behaviours, events or outcomes involve patient care, speaking up is mandated and enshrined within duty of candour and open disclosure laws and guidelines by multiple national professional bodies ( Stephenson, 2018 ).

Researchers have conceptualised the “continuum of disclosure” to include acts and signals involving interprofessional interactions ranging from silence and withholding, gossip and derision to speaking up and whistleblowing. All these disclosure acts with healthcare have associated social, cultural and organisational ramifications for patients, healthcare organisations and their employees ( Gagnon and Perron, 2020 ; Faÿ, 2008 ; Guendouzi, 2001 ). Mannion *et al.* note the need to explore whistleblowing as “an unfolding, situated and interactional process and not just a one-off act by an identifiable whistle-blower” ( Mannion *et al.* , 2018 ). While speaking up has been characterised as a relatively informal interprofessional act, it is nonetheless influenced by multiple, complex organisational and interpersonal factors ( Nacioglu, 2016 ; Preckel *et al.* , 2020 ; Okuyama *et al.* , 2014 ; Blenkinsopp *et al.* , 2019 ). In discussing the conceptual underpinnings of whistleblowing, Mannion *et al.* elaborate on the continuum of wrongdoing disclosure behaviours. They highlight that while there is a lack of clear distinction between the concepts of speaking up, raising concerns and whistleblowing, these terms are often used interchangeably. They also discuss the characteristics of processes of disclosure that inflect actions with differential implications. Mannion *et al.* assert that distinguishing between raising concerns, speaking up and whistleblowing involves considerations about: (1) formal or informal mechanisms used, (2) the progression of disclosure upwards along the chain of command, drawing the attention of increasingly senior internal professionals and (3) whether the disclosure was directed towards internal or external entities. For instance, a healthcare worker who discusses their misgivings with their line manager about quality and safety can be characterised as raising concerns. Speaking up implies usage of higher degree of formalised methods of disclosure that may involve registering or recording their concerns with immediate line managers or other interprofessional groups within the organisation. All actors involved in these forms of speaking up may share similar mental models for assertive communication, as well as common expectations about what action should follow these acts of disclosure. Whistleblowing involves the escalation of voicing concerns through formal channels to draw attention of a more senior internal or external entity such as an ombudsman or governing bodies and professional regulators to wrongdoing within the organisation. Acts of whistleblowing may have increased financial and legal ramifications for the organisations within which unprofessional behaviours have been observed and reported. Often, these actions of disclosure appear to progress along a continuum in relation to a perception of a lack of action on the part of the individual or entity with whom the employee raised their concerns in the first place ( Mannion *et al.* , 2018 ).

Just as important within the literature related to staff in healthcare organisations raising their concerns is the co-occurring reception of such disclosure through either individual, collective, or organisational learning, listening or deafness that ultimately determine organisational responses to speaking up ( Jones and Kelly, 2014 ). Key barriers to speaking up for safety in healthcare organisations are related to perceptions and experiences of an organisation's “just culture” or its lack thereof ( Khatri *et al.* , 2009 ; van Marum, 2022 ). Widespread fear of reprisal or retaliation, implications for team functioning, and perceptions related to the futility of speaking up are key themes that have been identified within the literature as factors that are barriers to speaking up ( Brown *et al.* , 2014 ; van Baarle *et al.* , 2022 ; Frankel, 2006 ). Evaluations of culture change interventions within hospital settings have identified the need to supplement speaking up skills and practices among staff, with accompanying changes across several related organisational and contextual factors ( McKenzie *et al.* , 2019 ; O'Donovan and McAuliffe, 2020 ; Ginsburg and Bain, 2017 ; Westbrook *et al.* , 2020 ; Gregory *et al.* , 2018 ; Jones *et al.* , 2021 ).

Staff demographics, clinical environments and interprofessional dynamics have been identified as important factors influencing whether staff speak up or remain silent, irrespective of their ability and skills to speak up ( Martinez *et al.* , 2017 ; Pattni *et al.* , 2019 ; Labrague and De los Santos, 2020 ; Jones and Durbridge, 2016 ; Westbrook *et al.* , 2020 ). Attention has been drawn to the need for further investigation into the psycho-social and cultural dimensions of speaking up, as well as the need for the integration of these dimensions into speaking up skills-training programmes in healthcare settings ( Kim *et al.* , 2020 ; Feeser *et al.* , 2021 ). When viewed within the context of organisational psychology, acts of individual or collective proactivity within hospital workplace systems, including acts of speaking up, might only be effective when they do not perturb the existing social and relational balance within an organisation ( Parker *et al.* , 2019 ). Against this background, our analysis employs a Bourdieusian lens to understand how multiple forms of personal and interpersonal psycho-social and cultural resources influence acts of speaking up among hospital staff as well as the structural, institutional and physical spaces within which these acts unfold.

## Symbolic forms of capital, socially co-constituted realities and organisational violence

In his seminal work “The Forms of Capital”, Pierre Bourdieu argued that “… it is … impossible to account for the structure and functioning of the social world unless one reintroduces capital in all its forms and not solely in the one form recognized by economic theory” ( Bourdieu, 1986 ). He developed the concepts of “social capital” and “cultural capital” as ways of explaining the symbolic value-laden representations, exchanges and artefacts that are co-constructed and laden with meaning within social groups. Bourdieu argued that through membership and participation within a group or network of individuals, through institutionalised and informal processes, inclusion and acceptance within the group entitles members to active or potential material or non-material resources such as mutual acquaintances, recognition, trust, care, solidarity and reciprocity. The form of resources that an individual possesses by virtue of membership and inclusion within a network can be conceptualised as social capital. Cultural capital refers to the symbolic elements that signify belonging to a particular social class. Forms of symbolic capital may include shared mannerisms, professional credentials, skills, shared educational backgrounds and professional positions. Other elements of symbolic capital can be signifiers of class, such as posture, tastes, clothing, mannerisms, material belongings, etc.

The multiple nodes within which power is concentrated within healthcare organisations has also been framed in terms of a “new class” theory through which forms of authority such as professional knowledge allow for symbolic domination and subjugation within healthcare settings ( Brewer, 1996 ). Therefore, the collective recognition of socially constituted notions within healthcare organisations, such as a belief that surgeons represent not just the functional role that they perform, but that their professional role also implies an associated higher economic reward, knowledge, expertise, professional prestige and access to influential networks, appears to confer on groups of professionals a form of “nobility” or symbolic power. The “habitus” within healthcare organisations, therefore, can be understood as the fabric, or a composite of material and symbolic exchanges, ways of being and collective co-constituted reality where the work of caring for patients unfolds ( Lo and Stacey, 2008 ). Further, this shared reality or “illusio” that healthcare employees occupy and co-create, continually shapes their learning ( Benozzo and Colley, 2012 ), their speech ( Canagarajah, 2020 ) and their emotional and social habitus ( Virkki, 2008 ) that then also informs their very disposition and personhood ( Daniels and Warmington, 2007 ). Therefore, these dynamics create a self-sustaining and reinforcing system over time and increased exposure can, in theory, be observed in the interactions of healthcare employees that are characterised within value-laden frameworks as constituting either professional or unprofessional behaviours.

Johanna Shapiro, in “Violence in medicine” discusses how Bourdieu's ideas related to “symbolic violence” can be applied in the context of organisational management practices and structures in the field of medicine ( Shapiro, 2018 ). Shapiro highlights that the structural acceptance and normalisation of “… ways that minimise or disrespect … (the) full humanity” of people within healthcare organisations can be understood as structural or organisational violence in hospitals. For example, medication errors or the refusal or inability of healthcare workers to “speak up” against professional misbehaviour is portrayed in binary terms of failure and success. Therefore, individual workers are implicated in these supposed failures while organisations practice a structural and “fundamental dishonesty” that is embedded in healthcare practices and systems.

Previous studies have used the concept of social capital in relation to safety culture to argue that healthcare organisational culture is heavily influenced by the social capital in an organisation which is reflected through the mutual trust and support demonstrated between employees ( Pfaff *et al.* , 2009 ). Therefore, the role of power relations within an organisation in conjunction with an employee's non-material forms of capital is central to how healthcare employees engage with one other, with organisational processes and how they respond to instances of wrongdoing that they believe may warrant speaking up or whistleblowing ( Mannion *et al.* , 2018 ).

## Situated subjecthood, normative violence and speaking up

The work of Kenny and Fotaki draws attention to the formative influence of embedded discourses in medical practice and institutions on the psyche of healthcare workers ( Kenny and Fotaki, 2019 , 2020 ). They propose the theory of “normative power and violence”, moving away from the framing of power as a distinct resource in healthcare organisations, particularly in reference to practices of disclosure of wrongdoing. They argue that the subjectivity of whistle-blowers is very much constituted and situated within the entrenched power dynamics in their organisations and the healthcare sector, in general. In combination with the Bourdeusian concepts of habitus, fields and symbolic forms of capital, the subjecthood of all human stakeholders within a healthcare organisation is therefore, influenced not just by formal structures, roles and processes, but also a range of intersectional psycho-social and cultural factors such as age, class, gender, ability (or disabilities), ethnicity, race, migration status, sexual orientation, linguistic skill and type of working arrangements and employment ( Worth, 2016 ; Fotaki, 2010 ; Silva, 2016 ; Tatli *et al.* , 2012 ; Tye-Williams *et al.* , 2020 ; McDermott, 2006 ; Rajendran *et al.* , 2017 ). The theory of normative power provides a useful framework to understand the psycho-social and cultural tensions experienced by employees who speak up against implicit group norms. Some of these organisational and professional norms may be disavowed in formal codes, policy and legislation such as code of conduct policies, anti-discrimination, anti-bullying and harassment laws. Nonetheless, when these acts do occur, they appear to be widely accepted as normative ways of professional being and functioning in healthcare. Wu *et al.* have examined the interplay between formal and informal structures, processes, practices and networks of professionals and how these dynamics impact speaking up through the lens of the “formal and informal organisation” ( Wu *et al.* , 2021 ). Ultimately, these factors, comprising relational, cognitive and structural elements influence the situated sensemaking among healthcare professionals that inflects how speaking up is enacted and responded to ( Lockett *et al.* , 2014 ).

The multitude of factors that contribute to the situated subjecthood (SS) of employees within hospitals may help to explain interesting findings in recent research that has elaborated on the pervasiveness of unprofessional behaviour in healthcare organisations, and consequently, speaking up. Unprofessional behaviours are any interpersonal and interprofessional behaviour that display disrespect to one or more persons involved in or impacted directly or indirectly in the interaction. Examples of such behaviours range from incivility such as rudeness, exclusion, ostracism and undermining to persistent bullying, harassment and assault. A multi-site study across seven hospitals in Australia found that hospital staff who self-reported speaking up skills also indicated a lower occurrence and negative impact of unprofessional behaviours ( Westbrook *et al.* , 2020 ). Based on the literature discussed above, this finding could possibly be explained through the lens of Bourdieu's theories. It is possible that the factors that influence an employee's ability, capacity and degree of comfort with speaking up are a composite of their symbolic capital and SS, differentiating how and whether employees speak up or remain silent. There is a paucity of research that examines the interplay between speaking up in healthcare organisations and personal and professional factors such as age, gender, length of employment within an organisation and social support from peers and superiors. Additionally, the relationship between speaking up and ongoing exposure to unprofessional behaviours resulting in enculturation or normalisation and acceptance of these behaviours among healthcare staff in Australia needs further study. Existing research appears to be limited to studies within professional groups such as nurses or medical students, and there is a need to examine whether these factors impact the speaking up behaviours and experiences of all staff within healthcare organisations, irrespective of their role within medical or non-medical fields, as all workers within healthcare environments contribute to the maintenance of organisational culture and speaking up efforts.

## Purpose

Our study aimed to map how the symbolic capital and SS of individual employees contribute to their capacity to speak up in the face of unprofessional behaviour within healthcare organisations. We aimed to do this by performing secondary analysis on the responses of over 5,000 hospital staff to a survey around negative workplace behaviours. Based on Bourdieu's definitions of categories such as social capital, cultural capital, we theorise that an employee's ability and decision to speak up will be influenced by multiple fixed demographic characteristics and their exposure to unprofessional and discriminatory behaviours within their workplace. Therefore, we hypothesise that certain individual characteristics might increase or decrease the potential of an employee's exposure to unprofessional behaviours, as well as influence their experience, perception of organisational support and their speaking up behaviours within their workplace. These characteristics include gender, class, age and demographic characteristics such as sexual orientation, carer responsibilities, ethnicity, race, disability etc. We use the term “situated subjecthood” (SS) to refer to this unique nature of professional personhood experienced by healthcare workers in relation to their workplace environments. We propose that an employee's SS in combination with their symbolic capital (SC, a combination of their social and cultural capital, which can also be thought of as their non-material capital), as indicated by their professional role, their length of employment, and therefore, familiarity with their hospital sites and other professionals will reflect employees' enactment, or lack thereof, of speaking up against unprofessional behaviour. Therefore, our research question is: How do the symbolic capital and SS of hospital staff impact speaking up?

## Methodology

### Design

The Longitudinal Investigation Of Negative behaviour (LION) survey was administered to hospital staff in seven hospitals across three Australian states as part of baseline data collection for a large-scale multi-site evaluation of a culture change intervention ( Westbrook *et al.* , 2020 ). The survey aimed to identify individual and organisational factors associated with unprofessional behaviours among hospital employees. All clinical and non-clinical staff members across the participating hospitals were invited to respond to questions about their experience of 26 types of unprofessional behaviours, ranging from rudeness to assault. Statements assessed frequency of exposure across a 7-point Likert scale (“never”, “1–2 times/year”, “every few months”, “around monthly”, “weekly”, “daily” and “multiple times daily”. Exposure to unprofessional behaviours as a target as well as, as a bystander were assessed with exposure frequencies mapped against “This has happened to me” and “I have seen this happen to someone else – patients, staff, visitors”. The survey also requested responses about staff perceptions related to speaking up. Two open-ended, optional questions invited respondents to share their comments on their experiences related to unprofessional behaviours. These questions were: “Are there any specific instances of unprofessional staff behaviours that you would like to describe?”, and “Are there any other comments you would like to make about staff behaviour in this hospital?”

### Analysis

The response rate to the survey was 34% ( *n* = 5,178) with proportional representation across professional groups. Primary analysis of the survey results showed that self-reported speaking up skills among hospital employees was strongly associated with a lower frequency of experiencing unprofessional behaviour as well as reduced negative impact ( Westbrook *et al.* , 2020 ).

Analysis of data for the secondary analysis reported in this article was carried out in two stages. Stage one involved analysing LION survey data quantitatively based on our theoretical framework to investigate how SC and SS of individual employees contribute to their capacity to speak up in the face of unprofessional behaviour within healthcare organisations. The second stage involved a qualitative analysis of narrative comments using an inductive approach. Narrative responses to two open-ended questions in the LION survey were extracted and analysed to understand how SS and symbolic capital of hospital staff influenced the experience of unprofessional behaviours as well as speaking up behaviours.

Results from both stages of the analyses are presented in relation to the two dimensions under investigation: SC and the individual enactment of speaking up; and SS and organisational culture.

The section of the LION survey related to speaking up measured respondents' level of agreement with the following statements on a five-point Likert scale, ranging from strongly agree to strongly disagree. Each of these statements was used to ascertain membership of hospital staff groups and perceptions or experiences that could be used as indicators of non-material resources of individual employees, such as social or cultural capital.

**Table:** 

Thematic indicators of non-material or symbolic capital	Statements related to speaking up beliefs, perceptions and experiences
Endorsement of safety culture – espoused belief	1. Speaking up or reporting unprofessional behaviours is important for patient safety
Indicator of social capital – support from colleagues	2. I am encouraged by my colleagues to speak up about unprofessional behaviour
Indicator of cultural capital – assertive communication skills	3. I have the skills to effectively speak up if I experience unprofessional behaviour
Indicator of cultural capital – assertive communication skills Indicator of social capital – affinity for social membership	4. I have the skills to effectively speak up if others experience unprofessional behaviour
Indicator of cultural capital – knowledge and accessibility to speaking up resources	5. I know the proper channels to raise concerns about unprofessional behaviour
Indicator of social capital – trust in organisational and social network	6. Unprofessional behaviour is effectively managed in this hospital
Indicator of cultural capital – psychological comfort with open and assertive communication	7. I feel comfortable speaking up or reporting unprofessional behaviour
Indicator of (inverse) cultural capital – depletion of non-material psychological and temporal resources	8. It takes too much time and effort to report unprofessional behaviour
Indicator of social capital – psychological confidence and trust in support from social and organisational network	9. I am confident I would receive support from my supervisor if I reported unprofessional behaviour
Indicator of (inverse) social and cultural capital – lack of psychological trust and fear about loss of professional credentials, development, membership, or progress	10. Speaking up or reporting unprofessional behaviour is likely to have a negative impact on my career
Indicator of social capital – psychological belief in support from social and organisational network	11. I am confident I would be believed and taken seriously if I reported unprofessional behaviour

Descriptive statistics were calculated (presented in the Appendix ) with accompanying data visualisation where applicable. All analyses were conducted using the MS Excel 2,106 statistical package. The findings reported in this article are in line with standards for reporting qualitative research (SRQR) ( O'Brien *et al.* , 2014 ).

Respondents who indicated that they agreed or strongly agreed to statements 2, 3, 4, 5, 6, 7, 9 and 11 and disagreed or strongly disagreed with statements 8 and 11 were grouped based on their stated professional role and similarity in operational tasks, role-specific training, managerial responsibilities etc. In addition to professional roles held by hospital employees, we also proposed that SS of hospital employees was a composite of their age, gender, their exposure to unprofessional behaviours over time and their length of employment at their workplace. We categorised temporal or time-based factors such as the age of staff and the length of time that they had been employed within the healthcare industry and within their hospital site and compared these categories in terms of their self-reported comfort to speak up (agreed or strongly agreed with statement 7). We also examined these data to understand whether gender played a role in how staff perceived and experienced speaking up. To explore the impact of exposure to unprofessional behaviours on the SS of staff, we extracted the responses of staff who had self-reported speaking up skills (agreed or strongly agreed with statement 3) and triangulated their responses against whether they felt comfortable speaking up against unprofessional behaviours (agreed or strongly agreed with statement 7) and whether they had ever witnessed or personally experienced any unprofessional behaviours. We compared this group of respondents against respondents who had never been exposed to these behaviours, neither as targets nor bystanders.

## Results

### Respondent characteristics

Of 5,178 responses to the survey, 93% of respondents ( *n* = 4,523) indicated that they believed speaking up was important for patient safety, indicating agreement and a sense of espousing safety culture principles. Establishing this shared belief allowed us to ascertain the validity of notions of “patient safety” and “speaking up” amongst respondents. The majority of respondents did agree with the centrality of speaking up and its role in promoting and maintaining patient safety.

In total, 93.7% of respondents ( *n* = 4,851) provided useable responses related to self-reported speaking up skills. In our analysis presented in this article, we investigated whether individuals' degree of comfort and capacity to speak up is influenced by their SC and SS. These categories are a composite of factors including, but not limited to age, length of employment with the healthcare sector, hospital site, gender, professional role and exposure to unprofessional behaviours. Narrative responses were provided by 28.6% staff ( *n* = 1,479) of 5,178 overall survey respondents. The characteristics of this sample of respondents appeared to be representative of the larger sample, and their demographic characteristics are presented in Table 1 below.

### Overview

Our findings indicate that both symbolic capital and SS intersectionally influence speaking up behaviours and experience of organisational culture among hospital staff. Professionals with higher degrees of symbolic capital have higher degrees of skills, capacity and support for speaking up against unprofessional behaviours. Factors that influence employees' decision to speak up and how they enact speaking up were: Seniority; increased professional status; internal social networks and associated supports, such as peer, management and senior professionals' support; internalised psychological beliefs about anticipated support and reception to disclosure; safety culture at the levels of professional groups and specialised training to improve interpersonal and individual psychological resilience, compassion and person-centred engagement.

SS factors such as length of employment within hospital sites as well as within the healthcare sector, age, gender, exposure to unprofessional behaviours impact how employees engage in speaking up about unprofessional behaviours. Comfort with speaking up declines with increased exposure to the hospital environment. Conversely, employment within the healthcare sector for longer periods of time in conjunction with increasing age of employees may enable staff to become more comfortable with speaking up over time. Gender also has an impact on speaking up, where male respondents reported higher individual speaking up capacity, confidence and organisational and peer support than other gender groups.

Our findings also demonstrated that a higher proportion of staff among those who had never been exposed to unprofessional behaviours were comfortable with speaking up as compared to staff who had been exposed to unprofessional behaviours. Unprofessional behaviours that actively diminished personhood such as discriminatory and unjust behaviours appeared to have a higher negative impact on the degree of comfort staff felt with speaking up, despite reporting that they had the skills to do so.

### Symbolic capital and the individual enactment of speaking up

**Figure 1:**
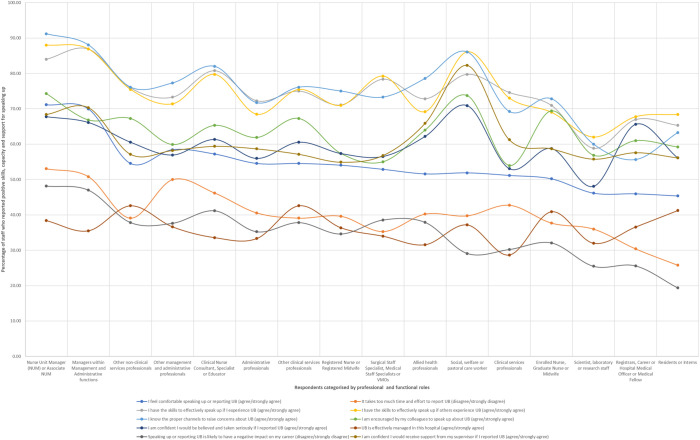
Visualisation of staff responses to statements related to speaking up based on professional roles

Comparatively higher percentages of staff from role categories with managerial responsibilities such as nursing unit managers (NUM) or associate NUMs and managerial staff within the management and administrative group appear to have higher self-reported individual capacity and ability to speak up against unprofessional behaviours, compared to other professional groups (e.g. residents and interns). Figure 1 (with data in Table A1 in the Appendix ) presents the grouping of respondents' positive responses to the speaking up statements within the survey. In addition, non-managerial professional groups whose roles demand higher degrees of person-centred care and empathy such as social, welfare or pastoral care workers responded with higher degrees of confidence in receiving peer support and encouragement. This group (social, welfare or pastoral care workers) also indicated the highest degree of confidence in supervisory support for speaking up against unprofessional behaviour. Other specialist staff who work in more socially isolated and individualised roles, such as scientists, laboratory and research staff and clinical services professionals (e.g. psychology, medical imaging, perfusionist, technologist and pathology collector) conversely showed the lowest percentage of staff who felt confidence around peer support or encouragement regarding speaking up against unprofessional behaviour. This finding points to the influence of social capital on speaking up against unprofessional behaviour. This group of specialists, along with groups within which there appears to be a higher representation of early to mid-career specialists (such as residents, interns and graduate nurses) had the highest percentage of staff who indicated that speaking up was likely to have a negative impact on their career.

Narrative accounts of speaking up by respondents referred to a variety of factors that influenced their perception of speaking up and their willingness, ability and approach to speaking up. Some respondents also commented on their perceptions of the efficacy and outcomes of speaking up that influence their decision for or against speaking up.

There is a strong belief that making a complaint will cause the whistle blower more problems and that the organization will close rank or sweep problems under the carpet, so that ***(hospital name) “brand” is not effected.- *Allied Health professional, male, aged 55–64*

The personal experience, perceptions and satisfaction of individual staff based on their engagement with remediation processes varied widely and influenced how they described their experiences of unprofessional behaviours and confidence in speaking up. Instances of witnessing retaliatory behaviour against whistle-blowers and those who have spoken up against unprofessional behaviours appear to solidify the internalised perception of the futility of speaking up.

There is a very clear and pervasive culture of bullying in this hospital which stems from the top levels of management and seeps down to the frontline workers … The bullying is in the form of veiled threats and, on occasion, outright accusations resulting in HR involvement, whether the accusations are based in truth or not … It seems to be directed to a certain few staff members who are on the “outer” as they have the temerity to question management … – *Clinical Nurse Consultant/Specialist/Educator, female, aged 45–54*

The cyclical reluctance to speak up, combined with a lack of confidence in the possibility of remedial action appears to erode employee satisfaction and belief in the organisation's ability to protect victims and take appropriate protective action when staff did speak up, or were subject to behaviour that compromised their wellbeing.

Comments from some employees indicated that there may be an overflow of existing negative interpersonal and organisational dynamics that influence how victimised employees interact with speaking up or reporting mechanisms, whether promoted as peer-reporting tools, or formal reporting tools.

… Sometimes it is difficult for people to discuss unprofessional behaviour as the person they may wish to complain about may be an ***(culture change program leader or occupy a leading role in) … the department – so they are effectively blocked from discussing a problem … *– Allied Health professional, female, aged 35–44*

Several respondents expressed the perception that there was organisational and leadership inaction, or conditional action, related to unprofessional behaviours. This could deter staff from speaking up against unprofessional behaviours irrespective of the severity and persistence of these behaviours.

One surgeon that we had as a patient, a few years ago now, was sexually verbally abusive to many staff members, including me, but nothing was done about it as the attitude was that he is a surgeon and therefore must be treated like a god. – *Allied Health professional, female, aged 35–44*

The converse of negative subcultures based on professional groups appeared to be true as well. This was evident in how staff from certain role groups such as social, welfare or pastoral care workers. Staff with specialised training aimed at developing empathy and compassion appeared to respond more favourably in their comments about the sub-cultures within their work groups. These categories of staff also indicated the highest comfort with speaking up, amongst all staff groups.

I work within a team who I can happily and proudly say treat each other … respectfully and professionally at all times … I feel working from a (trauma-informed) model with our clients washes over into our inter-staff relationships. – *Registered Nurse or Midwife, female, aged 45–54*

Confidentiality of reports and the impact upon those who do speak up was a frequent theme among commenters who spoke of their reluctance to report unprofessional behaviours. A few respondents noted that supervisors actively discouraged reporting of unsafe practices when staff had spoken up against unprofessional behaviours.

I have been directed by a senior executive to cease recording unsafe workload instances in ***(reporting mechanism) … I have been instructed to only make positive comments to *** surveyors, I have been instructed to not tell the truth about the issues we face to *** surveyors by senior executives in this organisation. *– Allied Health professional, female, aged 35–44*

Staff members who appear to be most at risk of being at the receiving end of unprofessional behaviours also appear to face barriers to speaking up resources and accessing reporting. This unequal accessibility to speaking up appears to perpetuate the maintenance of unequal remediation practices within the organisation.

Certain nurses in the ED bully the *** (support services) . I was called a fucking idiot by one, but nothing will be done about it because the nurse is friends with the manager. I cannot complain about staff on ***(reporting mechanism) because I do not know how to use a computer. I think it is unfair because people can complain about me using *** *(reporting mechanism)* but many of the (support staff) cannot say anything back due to our language and computer difficulties. – *Cleaner/environmental services, gender and age not disclosed*

Eventual escalation and reliance on formal and litigious routes for remediation appeared to be a last resort when staff felt disenfranchised and failed by existing protective mechanisms within their organisations.

I was told I would no longer be supported at work by my manager (I have nine diseases). I then consulted my lawyer friend who advised me to speak to the Dept of Human Rights. They advised me that my employer must make “reasonable adjustments” to accommodate my health issues. I then had a meeting with ***(senior management) who seemed unsympathetic but agreed to reinstate the minimal support I had previously received. This made my manager angry which made things more difficult at work … *– Allied Health professional, female, aged 35–44*

The intersectional nature of non-material capital with facets of personhood such as disability and gender appear to have compounded impacts on the speaking up behaviours of hospital staff.

### Situated subjecthood and organisational culture

**Figure 2:**
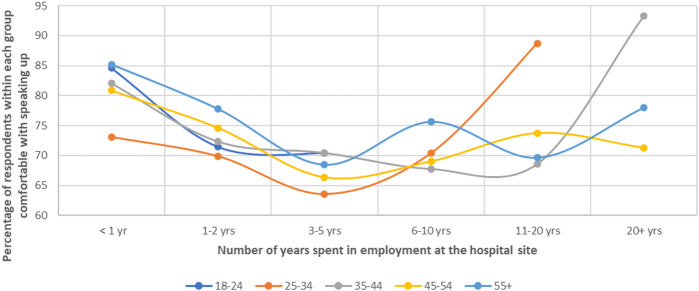
Respondents comfortable with speaking up based on age group and number of years spent in employment at their hospital site

For most age groups, it appears that comfort with speaking up declines with increased exposure to the hospital environment ( Figure 2 , Table A2 in Appendix ). This pattern could be attributed to the impact of enculturation within the workplace through exposure to unprofessional behaviours, thereby normalising and building tolerance for these behaviours. A sense of futility related to the process of speaking up as demonstrated by narrative comments may explain the finding around how longer periods of employment within the hospital site may temper employees' comfort with speaking up.

Outliers within our analysis may not be representative of larger employee groups. For instance, the increase in speaking up post 11 years of employment within the hospital site among 25–34-year-olds and for 35–44-year-olds post 20 years of employment is based on a small subset of employees. It is unlikely that there are many employees who fit these particular age and length of employment categories simultaneously, as indicated by our sample of only 18 ( *n* = 21, 85.71%) 25–34-year-olds who had worked between 11–20 years and 14 ( *n* = 15, 93.33%) 35–44-year-olds who had worked at the site for over 20 years. However, employment within the healthcare sector for longer periods of time in conjunction with increasing age of employees may be empower staff to become more comfortable with speaking up over time.

**Figure 3:**
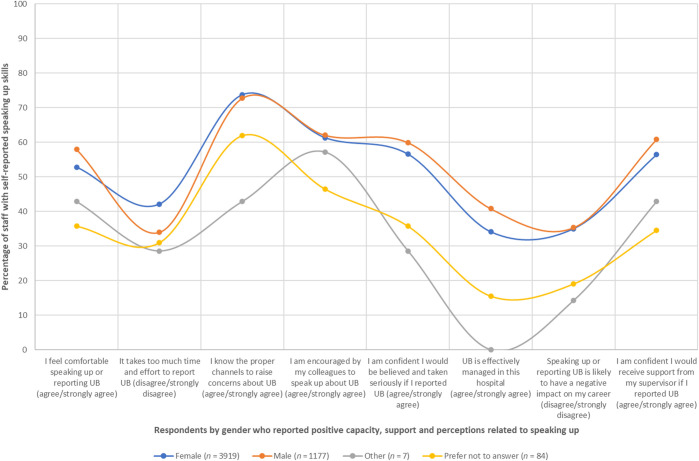
Visualisation of responses about self-reported speaking up skills, capacity and perceptions by gender

Gender also has an impact on subjecthood, where male respondents reported higher individual speaking up capacity, confidence and organisational and peer support. This appeared to enable more positive speaking up perceptions and experience among male staff, in comparison to women and people who identified with other gender categories ( Figure 3 and Table A3 in the Appendix ).

**Figure 4:**
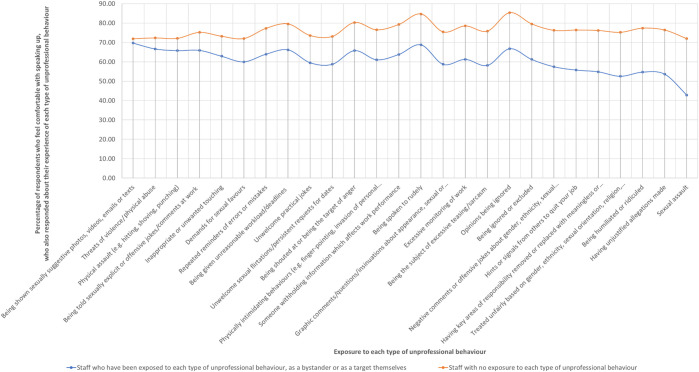
Respondents comfortable with speaking up who have never experienced and witnessed each of the 26 types of unprofessional behaviours (plotted in orange) against respondents who have been exposed to each of the unprofessional behaviours (plotted in blue)

To determine the relationship between exposure to unprofessional behaviours and comfort with speaking up, we mapped the percentage of staff who felt comfortable with speaking up while also having indicated that they had experienced or witnessed types of unprofessional behaviours. We compared this group of respondents against respondents who had never been exposed to these behaviours. Both groups of respondents had self-reported speaking up skills. Our findings demonstrated that a higher proportion of staff among those who had never been exposed to unprofessional behaviours were comfortable with speaking up as compared to staff who had been exposed to unprofessional behaviours ( Figure 4 and Table A4 in the Appendix ). In addition, behaviours that actively diminished personhood such as discriminatory and unjust behaviours appeared to have a higher negative impact on the degree of comfort staff felt with speaking up, despite reporting that they had the skills to do so. Therefore, we infer that exposure to unprofessional behaviour has a negative impact on the personhood of hospital staff who witness as well as experience any instances these behaviours first-hand.

Younger or junior staff, and staff who occupied roles that offered relatively lower symbolic capital and diminished subjecthood, appeared to not just be more vulnerable to experiencing unprofessional behaviour, but also struggled with feeling comfortable and conflicted about speaking up against these behaviours. A refusal to speak up in these situations appeared to indicate a recognition of the implications for the wrong-doer and sense of protection or loyalty, despite the personal cost and exposure to unprofessional behaviour endured.

I once had a staff member slap another staff member. Unfortunately, the younger staff member did not want to make a complaint, knowing it would almost certainly result in the older employee's termination. As a result, no action could be taken. *– Registered Nurse or Midwife, male, aged 25–34*

Despite being victimised by senior staff, the refusal to speak up by employees who have been at the receiving end of unprofessional behaviour highlights the burden of responsibility that is placed on victims to report events that may demand an exercise of social power and agency that they may not possess. Therefore, groups of professionals who had reported being vulnerable to unprofessional behaviours and negative organisational dynamics appeared to be not just the most impacted but also the least able to speak up for fear of negative personal or professional repercussions.

Several employees among vulnerable role, age and gender groups indicated an unwillingness to report unprofessional behaviours, despite self-reporting in the closed-ended questions the ability to speak up and having knowledge of reporting mechanisms.

I've seen the head of the one of the surgical departments being really demeaning to other doctors, including myself, at the unit meetings with 20+ other people present. His peers (i.e. other surgeons) are worried about saying anything to him, because he's very influential in the hospital and they will be targeted if they speak out. Which is how I feel too. – *Career/Hospital Medical Officer/Medical Fellow, female, aged 35–44*

While professional role and other demographic factors may be protective against experiencing unprofessional behaviour as well as providing additional individual capacity to speak up, exposure to unprofessional behaviours appeared to shift how staff perceived organisational culture and interacted within the workplace.

… a colleague and I were having a robust discussion and were not in agreement, so the person spoke louder to me, stepped forward and pointed their index finger close to my face. I asked them to step back, lower their voice and take their finger out of my face. They replied with, “but you don't understand” I replied I do understand but I don't agree, and this is what a robust discussion looks like. The person backed down and walked off shaking their head. This was all done within sight and hearing of three people in leadership roles at ***(hospital site), one asked if I was OK when it was over the other two didn't offer any support or acknowledgement of the staff member's bad behaviour. This not only altered the way I felt of the staff member but also of the three witnessing the behaviour. *– Nurse Unit Manager or Associate NUM, female, aged 45–54*

Exposure to unprofessional behaviours and the normalisation and acceptance of these behaviours within the organisation may therefore influence professional personhood and how staff experience and engage within their workplaces.

In combination with how personhood is co-created within healthcare workplaces, speaking up behaviours for multiple age, gender and staff groups are influenced by perceptions of organisational culture.

Employees' disillusionment with the organisational mechanisms that address unprofessional behaviour and their lack of confidence in leadership and management's commitment to employee well-being present a significant barrier to the willingness to report unprofessional behaviours. Lack of responsibility or the diffusion of responsibility for rectifying unprofessional appeared to create a vicious cycle of self-perpetuating negative sub-cultures, the misuse of power and unprofessional behaviours.

Power relations within each silo of disciplines … a clinician raising unprofessional behaviour of another clinician from a different discipline, reports up through line manager/stream manager only for the reporting clinician to be made “the problem” … shunt off to EAP ( *employee assistance programme* ) … unprofessional behaviour continues … Line manager and line manager's manager continually demonstrate unprofessional behaviour. HR dept also demonstrates unprofessional behaviour, no one else to report to, leads to isolation so the victims band together and share wounds for support so no resolution of the unprofessional behaviours. *Clinical Nurse Consultant / Specialist / Educator, female, aged 65+*

The weaponization of speaking up to further interpersonal conflicts may dilute the credibility of speaking up mechanisms and remediation tools intended to protect victims. Therefore, it appears that irrespective of the varied mechanisms for reporting and speaking up that are offered to employees, individual acts of speaking up are influenced by intersectional factors related to employee social capital and SS. These finding indicate that speaking up is inextricably linked to systemic and organisational dynamics that may perpetuate the prevalence of unprofessional behaviours.

## Discussion

The impacts of social and cultural capital and SS on speaking up in healthcare have not previously been assessed using a large-scale dataset of hospital staff. Our results demonstrate that a range of personal and professional factors impact the experiences, perceptions, skills, psycho-social capacity and ability of hospital staff to speak up when they experience or witness unprofessional behaviours. We had posited that the non-material resources of hospital staff members, namely their cultural and social capital experienced through the benefits that they give and receive via mutual acquaintances, recognition, trust, care, solidarity and reciprocity, play a role in enabling speaking up behaviours. These resources as argued by Bourdieu offer symbolic levers by which belonging and selfhood are formed, experienced and maintained.

Previous studies have highlighted the complexity of factors that impact the success of behaviour change interventions aimed at improving staff speaking up in hospitals ( Umoren *et al.* , 2021 ; Jones *et al.* , 2020 , 2021 ). Speaking up within healthcare organisations has been defined as a proactive act ( Parker *et al.* , 2019 ) of personal agency enacted by an individual healthcare worker on their own or another worker's behalf ( Kim *et al.* , 2020 ), through the use of written or verbal speech to arrest, deter and disrupt the prevalence of unprofessional behaviour that negatively impacts others within the hospital system ( O'Donovan and McAuliffe, 2020 ). Our study demonstrates the effect that age, gender, exposure to unprofessional behaviours, discrimination, enculturation through environmental immersion and organisational socio-cultural factors have on the beliefs, perceptions, experiences and behaviours of staff when they are faced with the prospect of speaking up against unprofessional behaviour. Based on these findings, we could argue that because of this effect, speaking up becomes a counter-cultural act within hospital environments, with inherently punitive implications for victims and bystanders, some more than others. Therefore, there is a tension between well-meaning culture change interventions that aim to reduce unprofessional behaviour within healthcare by promoting speaking up across hospitals, where pre-existing systemic and organisational inequities and socio-cultural patterns persist.

The development of the notion of positive psychological capital (PsyCap) as a composite of hope, optimism, resilience and self-efficacy (HOPE) as proposed by Luthans *et al.* may explain why certain employees may be protected against the negative impacts of unprofessional behaviour or otherwise toxic workplace cultures ( Luthans *et al.* , 2004 ). Within the literature related to psychological safety in the workplace, the link between social, cultural and psychological capital is often highlighted in terms of its impact on performance ( Santos *et al.* , 2018 ), learning ( Turner and Harder, 2018 ), job satisfaction ( Ommen *et al.* , 2009 ), work engagement and more recently, wellbeing ( Clausen *et al.* , 2019 ) and speaking up ( O'Donovan and McAuliffe, 2020 ). However, the burden of speaking up across all these bodies of work appears to be placed on victims within a system that is primed against them, in how it is structured to incentivise the very behaviours employees are expected to speak up against ( Pavithra, 2021 ). Therefore, Bourdieusian concepts of symbolic capital appear to have been reduced and misrepresented in practice among organisational improvement practitioners whose priorities are framed by narrowly defined metrics of profitability, risk avoidance or deflection and organisational productivity and efficiency ( Ernst *et al.* , 2018 ). The role of socio-cultural inequality within the workplace has often been re-framed as an area for individual intervention and increased personal resilience, rather than highlighting the need for wider organisational and systemic reform ( Hendy and Tucker, 2020 ).

We identified that certain staff groups appear to demonstrate a higher degree of confidence, comfort and intergroup support for speaking up even in the face of exposure to unprofessional behaviours. These groups appear to have access to specialised training that embeds non-technical skills such as providing person-centred care and trauma-informed care, where individual capacity for positive communication, empathy, compassion and equity are highlighted. This finding is unique and has not been reported by previous studies. Replicating such training across multiple staff groups within may have the potential to serve as a supplement to improve outcomes from speaking up and culture change interventions.

Our study adds to the existing literature that provides evidence around the centrality of organisational environments on withholding voice and speaking up behaviours among staff ( Schwappach and Richard, 2018 ). The overarching themes of our findings indicate that the impact of organisational culture, persistent exposure to unprofessional behaviour and extended systemic inequalities can significantly limit the capacity of employees speaking up against unprofessional behaviour.

## Limitations

The analysis of speaking up behaviours using data collected through the LION survey was performed upon finding a correlation in experience of unprofessional behaviours and speaking up behaviours from our primary analysis. This phased approach to analysis was undertaken as our primary findings warranted further investigation. Therefore, our application of Bourdieusian theory and modelling of dynamics within hospital systems was undertaken post initial analysis, rather than being built in during the research design process. We considered Bourdieusian theory the most suitable lens to elicit further insights around the effect we saw within our research sample – where symbolic capital appeared to have an impact on staff experience of unprofessional behaviours, as well as the impact these behaviours appeared to have on speaking up. A gap in our study design may have resulted from the decision to perform analysis on our data after primary analysis, rather than including more specifically designed questions that corresponded more directly against Bourdieusian theory. Therefore, our study does not address factors such as race, class, ethnicity, nationality, residential location, immigration status, self-identified cultural identity and type of employment that may indicate economic precarity (casual, contract work arrangements). We were limited to ascertaining these categories from open-ended comments or responses to questions related to discrimination. Survey instruments used to gauge perceptions, experiences and behaviours of staff rely on self-reporting, which may not always capture the breadth of experiences or the accuracy of phenomenon that respondents report on. Therefore, further studies that are designed to explore the relationships between symbolic capital, unprofessional behaviours and speaking up in healthcare systems, using observational and interview techniques can further clarify and validate the patterns that we have identified within this study.

## Conclusion

Our study has demonstrated that theoretical constructs such as symbolic capital and SS may have material impacts on how effective or sustainable culture change interventions can be within hospital environments.

Speaking up within healthcare organisations is not merely an individual undertaking but a result of multiple interactions between individual and collective actors within the hospital system. For culture change programmes that emphasize the need for greater reporting of unprofessional behaviours to be successful, hospital administrators and management need to consider the cyclical socio-cultural and psychological frameworks that influence speaking up behaviours among staff. Organisations appear to disable staff on the one hand, by being complicit in abusive subcultures and perpetuating cycles of disempowerment among staff through inaction, while on the other hand, encouraging victims to break rank by speaking up. Ultimately, organisations need to take note that employees with higher degrees of autonomy and enhanced psycho-social skills may feel more comfortable to speak up despite being exposed to unprofessional behaviours and negative organisational sub-cultures. Therefore, healthcare organisations might be able to improve the success of culture change interventions by replicating the training and employment conditions currently offered only to some employees, and extending these to all staff, particularly those who are most vulnerable and at risk of being subject to unprofessional behaviours. Through our study, we demonstrated that there are inherent limitations to speaking up that staff members experience as a function of how their professional personhood is formed within the healthcare organisations where they work. Therefore, we conclude that culture change interventions that promote speaking up as a significant mechanism to disrupt unprofessional behaviour in hospitals may be limited in their effectiveness. Efficacy of culture change interventions that use speaking up mechanisms could potentially be improved by supplementing these interventions with efforts to reduce systemic inequality, improve protections for staff who do speak up, ensure remediation and accounting for intersectional socio-cultural factors that may impact speaking up behaviours among staff.

## Figures and Tables

**Table 1:** Narrative comments respondents' demographic characteristics

Characteristics of staff who provided narrative comments used within this study			Overall survey respondents' characteristics
Gender	Male	287 (19.41%)	1,176 (22.71%)
	Female	1,154 (78.03%)	3,909 (75.49%)
	Other/not specified	38 (2.57%)	93 (1.80%)
Age	18–24	82 (5.54%)	300 (5.79%)
	25–34	397 (26.84%)	1,567 (30.26%)
	35–44	317 (21.43%)	1,127 (21.77%)
	45–54	332 (22.45%)	1,097 (21.19%)
	≥55	315 (21.30%)	983 (18.98%)
	Not specified	36 (2.43%)	104 (2.00%)
Role types*	Medical	166 (11.22%)	546 (10.54%)
	Nursing	674 (45.57%)	2,248 (43.41%)
	Allied health	242 (16.36%)	795 (15.35%)
	Support services	136 (9.20%)	590 (11.39%)
	Management and administration	245 (16.57%)	822 (15.87%)
	Not specified	16 (1.08%)	177 (3.42%)

**Note(s):** *Role types:

*Clinical*

Medical: Medical Staff Specialist/VMO, Registrar, Resident, Surgical/Anaesthetic Staff Specialist/VMO, Career/Hospital Medical Officer/Medical Fellow, Intern

Nursing: Nurse Unit Manager or Associate NUM, Registered Nurse or Midwife, Graduate Nurse or Midwife, Enrolled Nurse, Clinical Nurse Consultant/Specialist/Educator

Allied Health and Clinical Services: Allied Health, Clinical Services, Social, Welfare or Pastoral Care Worker

*Non-clinical*

Management and Administration: Ward Clerk/Patient Services Clerk, Administrative Staff, Manager, Other Administrative or Managerial Roles

Support Services: Personal Care/Patient Services Assistant or Orderly, Cleaner/Environmental Services, Other Support Services Staff, Food Services, Engineering Services, Security or Tradesperson, Scientist, Laboratory or Research Staff

**Table A1:** Hospital staff perceptions and experience related to speaking up based on professional roles

Speaking up enablers by role^*^		A	B	C	D
Individual	Comfort	179 (70.75%)	129 (68.25%)	334 (66.69%)	207 (57.98%)
	Time and effort	135 (53.36%)	94 (49.74%)	231 (45.65%)	175 (49.44%)
	Speaking up skills for self	210 (84%)	159 (86.89%)	406 (79.76%)	255 (73.28%)
	Bystander speaking up skills	220 (88%)	159 (86.89%)	384 (75.59%)	247 (71.39%)
	Knowledge of channels	228 (91.20%)	162 (88.04%)	390 (76.77%)	269 (77.30%)
Social	Colleagues' encouragement	182 (74.29%)	123 (66.85%)	332 (64.97%)	209 (59.89%)
	Being believed	166 (67.76%)	121 (66.12%)	319 (63.93%)	197 (56.94%)
Organisation	Effective management	96 (38.40%)	65 (35.52%)	251 (50.50%)	126 (36.63%)
	No fear of negative career impact	117 (48.15%)	86 (46.99%)	197 (39.17%)	131 (37.64%)
	Supervisor support	171 (68.40%)	128 (70.33%)	325 (64.36%)	202 (58.21%)

Speaking up enablers by role^*^		E	F	G	H
Individual	Comfort	231 (56.62%)	133 (54.29%)	60 (53.57%)	686 (53.76%)
	Time and effort	185 (45.45%)	99 (40.08%)	45 (40.18%)	507 (40.05%)
	Speaking up skills for self	319 (80.76%)	177 (72.24%)	84 (75%)	872 (71.07%)
	Bystander speaking up skills	314 (79.70%)	167 (68.44%)	83 (75.45%)	872 (70.95%)
	Knowledge of channels	323 (81.98%)	175 (71.72%)	86 (76.11%)	927 (75%)
Social	Colleagues' encouragement	258 (65.32%)	151 (61.89%)	74 (67.27%)	702 (57.35%)
	Being believed	241 (61.32%)	136 (55.97%)	66 (60.55%)	702 (57.35%)
Organisation	Effective management	132 (33.59%)	79 (33.33%)	46 (42.59%)	440 (36.36%)
	No fear of negative career impact	161 (41.18%)	86 (35.25%)	42 (37.84%)	424 (34.61%)
	Supervisor support	234 (59.39%)	142 (58.68%)	64 (57.14%)	672 (54.90%)

Speaking up enablers by role^*^		I	J	K	L
Individual	Comfort	111 (50.92%)	226 (51.02%)	42 (52.50%)	69 (51.11%)
	Time and effort	77 (35.98%)	177 (39.78%)	31 (38.75%)	59 (43.38%)
	Speaking up skills for self	163 (78.37%)	319 (72.83%)	63 (79.75%)	94 (74.60%)
	Bystander speaking up skills	164 (79.23%)	303 (69.18%)	68 (86.08%)	92 (73.02%)
	Knowledge of channels	151 (73.30%)	345 (78.59%)	69 (86.08%)	90 (69.23%)
Social	Colleagues' encouragement	115 (55.02%)	279 (63.99%)	59 (73.75%)	68 (53.97%)
	Being believed	117 (56.52%)	273 (62.19%)	56 (70.89%)	69 (53.08%)
Organisation	Effective management	69 (33.99%)	138 (31.58%)	29 (37.18%)	37 (28.68%)
	No fear of negative career impact	81 (38.57%)	166 (37.90%)	23 (29.11%)	39 (30.23%)
	Supervisor support	117 (56.80%)	288 (65.90%)	65 (82.28%)	79 (61.24%)

Speaking up enablers by role^*^		M	N	O	P
Individual	Comfort	127 (50.4%)	25 (45.45%)	57 (45.60%)	45 (45.92%)
	Time and effort	96 (38.25%)	18 (33.96%)	38 (30.16%)	25 (25.51%)
	Speaking up skills for self	171 (70.95%)	30 (58.82%)	83 (66.94%)	64 (65.31%)
	Bystander speaking up skills	167 (69.01)	31 (62%)	84 (67.74%)	67 (68.37%)
	Knowledge of channels	177 (72.84%)	30 (60%)	69 (55.65%)	62 (63.27%)
Social	Colleagues' encouragement	168 (69.42%)	29 (56.86%)	75 (60.98%)	58 (59.18%)
	Being believed	144 (58.78%)	25 (48.08%)	82 (65.60%)	55 (56.12%)
Organisation	Effective management	99 (40.91%)	16 (32%)	45 (36.59%)	40 (41.24%)
	No fear of negative career impact	78 (32.10%)	13 (25.49%)	32 (25.60%)	19 (19.39%)
	Supervisor support	142 (58.68%)	29 (55.77%)	72 (57.60%)	55 (56.12%)

**Note(s):** * Professional role groups:

A. Nurse Unit Manager (NUM) or Associate NUM

B. Managers within Management and Administrative functions

C. Other non-clinical services professionals

D. Other management and administrative professionals

E. Clinical Nurse Consultant, Specialist or Educator

F. Administrative professionals

G. Other clinical services professionals

H. Registered Nurse or Registered Midwife

I. Surgical Staff Specialist, Medical Staff Specialists or VMOs

J. Allied health professionals

K. Social, welfare or pastoral care worker

L. Clinical services professionals

M. Enrolled Nurse, Graduate Nurse or Midwife

N. Scientist, laboratory, or research staff

O. Registrars, Career or Hospital Medical Officer or Medical Fellow

P. Residents or Interns

**Table A2:** Respondents with self-reported speaking up skills (for themselves and as a bystander), who also agreed or strongly agreed with the statement “I feel comfortable speaking up or reporting unprofessional behaviour” categorised by age and employment duration

Respondents comfortable with speaking up grouped by age group against number of years employed at the hospital site						
	<1 yr ( *n* = 442)	1–2 yrs ( *n* = 495)	3–5 yrs ( *n* = 674)	6–10 yrs ( *n* = 647)	11–20 yrs ( *n* = 658)	20+ yrs ( *n* = 330)
18–24 ( *n* = 169)	66 (84.62%, *n* = 78)	45 (71.43%, *n* = 63)	19 (70.37%, *n* = 27)	0 ( *n* = 1)	0	0
25–34 ( *n* = 920)	155 (73.11%, *n* = 212)	158 (69.91%, *n* = 226)	173 (63.6%, *n* = 272)	133 (70.37%, *n* = 189)	18 (85.71%, *n* = 21)	0
35–44 ( *n* = 715)	64 (82.05%, *n* = 78)	68 (72.34%, *n* = 94)	124 (70.45%, *n* = 176)	124 (67.76%, *n* = 183)	116 (68.64%, *n* = 169)	14 (93.33%, *n* = 15)
45–54 ( *n* = 735)	38 (80.85%, *n* = 47)	50 (74.63%, *n* = 67)	71 (66.36%, *n* = 107)	107 (69.03%, *n* = 155)	180 (73.77%, *n* = 244)	82 (71.3%, *n* = 115)
55+ ( *n* = 707)	23 (85.19%, *n* = 27)	35 (77.78%, *n* = 45)	63 (68.48%, *n* = 92)	90 (75.63%, *n* = 119)	156 (69.64%, *n* = 224)	156 (78%, *n* = 200)

**Table A3:** Responses of staff by gender to self-reported speaking up questions grouped according to how they reflected individual, social and organisational enablers to speak up

Speaking up enablers		Female ( *n* = 3,919)	Male ( *n* = 1,177)	Other ( *n* = 7)	Prefer not to answer ( *n* = 84)
Individual	Comfort	2,070 (52.82%)	682 (57.94%)	3 (42.86%)	30 (35.71%)
	Time and effort	1,651 (42.13%)	400 (33.98%)	2 (28.57%)	26 (30.95%)
	Knowledge of channels	2,891 (73.77%)	856 (72.73%)	3 (42.86%)	52 (61.9%)
Social	Colleagues' encouragement	2,403 (61.32%)	730 (62.02%)	4 (57.14%)	39 (46.43%)
	Being believed	2,217 (56.57%)	705 (59.9%)	2 (28.57%)	30 (35.71%)
Organisation	Effective management	1,335 (34.06%)	480 (40.78%)	(0%)	13 (15.48%)
	No fear of negative career impact	1,372 (35.01%)	416 (35.34%)	1 (14.29%)	16 (19.05%)
	Supervisor support	2,211 (56.42%)	716 (60.83%)	3 (42.86%)	29 (34.52%)

**Table A4:** Staff with self-reported speaking up skills who felt comfortable with speaking up despite being exposed to different types of unprofessional behaviour compared against staff who responded as never having experienced these types of behaviours and who also reported that they felt comfortable with speaking up

Type of unprofessional behaviour (unprofessional behaviour)	Staff with self-reported speaking up skills and who agreed or strongly agreed with the statement “I feel comfortable speaking up or reporting unprofessional behaviour” and …	
	… who have been exposed to type of unprofessional behaviour, as a bystander or as a target themselves	… with no exposure to type of unprofessional behaviour
Being shown sexually suggestive photos, videos, emails, or texts	69 (69.7%, *n* = 99)	2,148 (71.89%, *n* = 2,988)
Threats of violence/physical abuse	106 (66.67%, *n* = 159)	2,036 (72.3%, *n* = 2,816)
Physical assault (e.g. hitting, shoving, punching)	50 (65.79%, *n* = 76)	2,115 (72.09%, *n* = 2,934)
Being told sexually explicit or offensive jokes/comments at work	501 (65.92%, *n* = 760)	1,519 (75.16%, *n* = 2,021)
Inappropriate or unwanted touching	105 (62.87%, *n* = 167)	2,017 (73.16%, *n* = 2,757)
Demands for sexual favours	6 (60%, *n* = 10)	2,259 (71.99%, *n* = 3,138)
Repeated reminders of errors or mistakes	624 (63.93%, *n* = 976)	1,098 (77.22%, *n* = 1,422)
Being given unreasonable workload/deadlines	1,081 (66.2%, *n* = 1,633)	901 (79.52%, *n* = 1,133)
Unwelcome practical jokes	203 (59.53%, *n* = 341)	1,873 (73.54%, *n* = 2,547)
Unwelcome sexual flirtations/persistent requests for dates	37 (58.73%, *n* = 63)	2,104 (73.08%, *n* = 2,879)
Being shouted at or being the target of anger	885 (65.8%, *n* = 1,345)	862 (80.26%, *n* = 1,074)
Physically intimidating behaviours (e.g. finger-pointing, invasion of personal space, blocking)	444 (61.07%, *n* = 727)	1,495 (76.47%, *n* = 1,955)
Someone withholding information which affects work performance	836 (63.82%, *n* = 1,310)	1,098 (79.22%, *n* = 1,386)
Being spoken to rudely	1,652 (68.8%, *n* = 2,401)	254 (84.67%, *n* = 300)
Graphic comments/questions/insinuations about appearance, sexual or private life	246 (58.71%, *n* = 419)	1,805 (75.4%, *n* = 2,394)
Excessive monitoring of work	576 (61.28%, *n* = 940)	1,142 (78.49%, *n* = 1,455)
Being the subject of excessive teasing/sarcasm	268 (58.13%, *n* = 461)	1,678 (75.82%, *n* = 2,213)
Opinions being ignored	1,393 (66.78%, *n* = 2,086)	546 (85.31%, *n* = 640)
Being ignored or excluded	674 (61.22%, *n* = 1,101)	1,141 (79.51%, *n* = 1,435)
Negative comments or offensive jokes about gender, ethnicity, sexual orientation, religion, disability, pregnancy, parenting responsibilities	250 (57.47%, *n* = 435)	1,731 (76.22%, *n* = 2,271)
Hints or signals from others to quit your job	179 (55.76%, *n* = 321)	1,686 (76.36%, *n* = 2,208)
Having key areas of responsibility removed or replaced with meaningless or unpleasant tasks	209 (54.86%, *n* = 381)	1,823 (76.15%, *n* = 2,394)
Treated unfairly based on gender, ethnicity, sexual orientation, religion, disability, pregnancy, parenting responsibilities	145 (52.54%, *n* = 276)	1,864 (75.19%, *n* = 2,479)
Being humiliated or ridiculed	274 (54.69%, *n* = 501)	1,653 (77.35%, *n* = 2,137)
Having unjustified allegations made	214 (53.63%, *n* = 399)	1,726 (76.37%, *n* = 2,260)
Sexual assault	3 (42.86%, *n* = 7)	2,251 (71.94%, *n* = 3,129)
